# Antioxidant and α-glucosidase inhibitory activities of compound isolated from *Stachytarpheta jamaicensis* (L) Vahl. leaves

**DOI:** 10.1038/s41598-023-45357-z

**Published:** 2023-10-30

**Authors:** Sri Fatmawati, Fithrotul Auwaliyah, Yuliana, Nur Hasanah, Devi Anggraini Putri, Healthy Kainama, Muhammad Iqbal Choudhary

**Affiliations:** 1 Department of Chemistry, Faculty of Science and Data Analytics, Institut Teknologi Sepuluh Nopember, Jawa Timur, Surabaya, 60111 Indonesia; 2 Department of Medical Laboratory Technology, Sekolah Tinggi Ilmu Kesehatan Ngudia Husada Madura, Bangkalan, 69116 Indonesia; 3 Laboratory of Chemistry, Chemistry Education Study Program, Faculty of Teacher Training and Educational Sciences, Pattimura University, Ambon, 97234 Indonesia; 4 H.E.J. Research Institute of Chemistry, International Center for Chemical and Biological Sciences, University of Karachi, Karachi, 75270 Pakistan

**Keywords:** Preventive medicine, Screening

## Abstract

*Stachytarpheta jamaicensis* is one of the folk medicines used for the treatment of diabetes in Ambon, Indonesia, but there are limited studies on the bioactivities of its constituents. This study aims to assess the antioxidant and antidiabetic activities of four extracts of *S. jamaicensis* leaves extracted using several solvents. Bioassay guided fractionation on each extract establishes for exploring *S. jamaicensis* leaves active compounds*.* The antioxidant was evaluated using the DPPH and ABTS methods, while the α-glucosidase inhibitory was carried out in vitro assay. The results showed that the methanol extract of *S. jamaicensis* leaves displays inhibition of DPPH, ABTS and α-glucosidase activity compared to other solvent extracts. Furthermore, 6β-hydroxyipolamiide was successfully isolated from the methanol extract of *S. jamicensis* leaves which was reported to have α-glucosidase inhibitory activity with an IC_50_ of 539.17 μg/mL. Based on the results, *S. jamaicensis* could be recommended as an antioxidant and antidiabetic agent.

## Introduction

For centuries, several herbal plants around the world have been used as a source of exogenous antioxidants^1, 2^, which have the ability to inhibit free radicals. Furthermore, free radicals are compounds with at least one unpaired electron^3, 4^ and elevated levels in the body can increase susceptibility to various diseases. This condition often leads to oxidative stress, which is one of the main causes of diabetes complications induced by hyperglycemia. Several studies showed that hyperglycemia is characterized by increased production of glycosidases and free radicals, thereby altering enzymes or non-enzymatic antioxidant defenses. Oxidative stress can also increase mitochondrial DNA damage as well as cause axonal cell death, leading to neuropathy^5^.

α-Glucosidase (EC 3.2.1.20) is an enzyme located in the small intestine, where it catalyzes the release of glucose from complex carbohydrates^6^. The glucose released has been linked to the occurrence of postprandial hyperglycemia in patients with diabetes mellitus. Furthermore, α-glucosidase inhibitor is antihyperglycemic agents that can be used to prevent postprandial hyperglycemia and they include acarbose, voglibose and miglitol^7^. Apart from the inhibitor, several natural resources and isolated compounds have been reported to have similar effects, including lanostane-type triterpenoids from *Ganoderma lucidum*^6^ as well as flavanones from *Chromolaena odorata*^8^. Based on these findings, the discovery of new α-glucosidase inhibitor from natural products is valuable for the prevention and treatment of postprandial hyperglycemia. Over the years, more than 400 plant species have been found to have antidiabetic activity. However, the search for new antidiabetic drugs from plants continues because they contain various types of secondary metabolites that show alternative and safe effects for patients. These secondary metabolites are often in the form of glycosides, alkaloids, terpenoids, flavonoids and others^9^.

*Stachytarpheta jamaicensis* (*Verbenaceae*), also known as kakurang or pecut kuda in Indonesia, is predominantly found in the tropical and subtropical regions of Asia, Africa and Oceania^10^. A previous ethnobotanical study showed that the local people in Ambon-Indonesia commonly used this plant as the traditional medicine for wound healing in diabetic patients. Liew and Yong (2016) also reported that it has a wide range of bioactive phytochemical compositions, suggesting its potential for healthcare. Furthermore, previous studies showed the bioactivities of *S. jamaicensis* as an antioxidant^11, 12^, antimicrobial^13^, immunomodulatory effect^14^ and antidiarrheal agent^15^. Apart from these activities, it has also been reported to have anti-hyperglycemic effects. This was evident by the ability of its methanolic extracts^16^ to significantly decrease blood glucose levels among streptozotocin-induced diabetic test rats. Another study showed that the ethanolic leaves extract caused a significant reduction in glycemic levels in alloxan-induced diabetic Sprague Dawley rats^12^. The methanolic extract of *S. jamaicensis* was shown to exhibit strong antioxidant activity with an IC_50_ of 5.0 µg/mL, but ascorbic acid as a standard drug had a higher value at 9.0 µg/mL^17^. These findings suggested that the plant could serve as an antioxidant and antidiabetic agent, but there were no studies on the bioactivities of its isolated compound. Therefore, this study aims to assess the antioxidant and antidiabetic activities of a new phthalate isolated from *S. jamaicensis* using the DPPH method and an in vitro α-glucosidase inhibitory assay.

## Methods

### Chemicals and reagents

Organic solvents used in this study were ethyl acetate, *n*-hexane, methanol, dichloromethane and ethanol. Other chemicals included dimethylsulfoxide (DMSO) (Merck), rat intestinal acetone powder (Sigma, 1639), 2,2’-azinobis- (3-ethylbenzothiazoline-6-sulfonic acid) (ABTS) (Wako), glucose kit liquor (HUMAN), potassium peroxydisulphate (K_2_S_2_O_8_) and 2,2-diphenyl-1-picryl-hydrazly (DPPH) (TCI, 1898-66-4). The positive control consisted of acarbose, gallic acid and Trolox (Wako). Furthermore, silica gel 60 G, 60 and 60 F_245_ aluminum sheets (Merck) were utilized for the fractionation process. 6β-hydroxyipolamiide was isolated from the methanolic extract of *S. jamaicensis* leaves^18^.

### Plant material

*S. jamaicensis* leaves were collected at Ambon Island, Indonesia in August 2017. The plant in this study is a wild plant. The collection of *S. jamaicensis* leaves and experimental research has complied with relevant institutional, national, and international guidelines and legislation. Then, the samples were taken to the Laboratory of Fundamental Biology, Pattimura University to be identified and labeled as specimen number 47. The identification of the plant samples was carried out by the Head of the Laboratory of Fundamental Biology. The collection of *S. jamaicensis* does not require special permission.

### Extraction

*S. jamaicensis* leaves were dried at room temperature and ground into powder. The dried powder of *S. jamaicensis* leaves (30 g) was macerated with various solvents (200 ml) separately including *n*-hexane, ethyl acetate, dichloromethane and methanol. The solvent was evaporated using a rotary vacuum evaporator to obtain concentrated extracts, namely methanol (1.21 g), ethyl acetate (0.60 g), dichloromethane (0.69 g) and *n*-hexane (1.18 g) with yields of 4.03, 2.00, 2.30 and 3.39% respectively. The extraction process was carried out to evaluate their antioxidant and diabetic activities.

### Antioxidant activity

#### DPPH radical scavenging assay

The capability of *S. jamaicensis* leaves extracts, including *n*-hexane, dichloromethane, ethyl acetate, methanol and 6B-hydroxyipolamiide, to scavenge DPPH radical was assessed based on a predetermined method^8^. The test solution was obtained from the dissolution of the extract in methanol with a concentration of 10 mg/mL. Furthermore, the reaction mixture consisted of 1 mL DPPH solution 6 × 10^–5^ M, which was mixed with 33 µL of extract solution. The working solution was incubated for 20 min at room temperature (37 °C), followed by measurement of the absorbance using a UV–Vis spectrophotometer at 517 nm. A blank sample containing 33 µL of methanol in DPPH solution was prepared and the absorbance was measured at the same wavelength. The positive control used for the assay system in this study was gallic acid. The DPPH radical scavenging activity (%) was determined using the Eq. (1).

1 $$\mathrm{Antioxidant \,activity }\left(\mathrm{\%}\right)=\left[\frac{\mathrm{Ab }-\mathrm{As}}{\mathrm{Ab}}\right]\times 100$$

where: Ab = the absorbance of DPPH radical, As = the absorbance of sample + DPPH radical

#### ABTS radical scavenging assay

ABTS cation radical was prepared by reacting ABTS (5 mL, 7 mM) with K_2_S_2_O_8_ (88 µL, 140 mM) and the solution produced was incubated at room temperature (37 °C) for 12–16 h. Subsequently, ABTS solution obtained from the process was solid blue and the absorbance was measured at 734 nm. Based on the predetermined standard, the absorbance before usage should be 0.7 ± 0.02. To obtain this value, the solution was diluted through the addition of ethanol.

Evaluation of antioxidant activity from *S. jamaicensis* leaves extracts (methanol, ethyl acetate, dichloromethane and* n*-hexane) was carried out by reacting 10 µL samples with 1000 µL ABTS radical solution. The mixture was then incubated at room temperature for 4 min and the absorbance was measured using a UV–Vis spectrophotometer at λ = 734 nm. Trolox was used as a positive control and ABTS radical scavenging activity (%) was calculated using Eq. (1).

#### In vitro alpha-glucosidase inhibitory assay

The antidiabetic potential of 6B-hydroxyipolamiide was evaluated using an in vitro α-glucosidase inhibitory assay based on a previous study. The enzyme supernatant was obtained from 1.00 g of rat intestinal acetone powder through centrifugation at 12,000 rpm for 30 min at 4 °C in 30 mL of normal saline. Furthermore, the working solutions were prepared by mixing 10 μL samples solution, 30 μL of 0.1 M buffer with pH 6.9, 20 μL of 10 mM maltose, 80 μL glucose liquor and 20 μL enzyme supernatant. They were then incubated for 10 min and the absorbance was measured with a concentration of 625 μg/mL using the Biotek ELx800UV microplate reader at 490 nm. The inhibitory activity was determined using the formula (2).

2 $$\mathrm{Inhibitory }\left(\mathrm{\%}\right)=\left(\frac{(\mathrm{Ablank}-\mathrm{Asample}}{\mathrm{Ablank}}\right)\times 100$$

where: Absorbansi_blank_ = A_enzyme reaction_ − A_blank of enzyme reaction_. Absorbansi_sample_ = A_sample reaction_ − A_blank of sample reaction_

### Fractionation and elucidation

The dried powder of *S. jamaicensis* leaves (3840 g) was extracted with methanol (19 L) for 3 × 24 h using the maceration method. The macerate of *S. jamaicensis* leaves was concentrated with a rotary vacuum evaporator and a crude extract of *S. jamaicensis* leaves was obtained. The methanol extract of *S. jamaicensis* leaves (100 g) was partitioned with an eluent ratio of *n*-hexane: methanol (6:1) to obtain methanol and *n*-hexane fractions. The methanol fraction (40 g) was fractionated using vacuum column chromatography with with a solvent ratio of ethyl acetate: acetone (100:0; 0:100) as the eluent to obtain five fractions (M1-M5). The M3 fraction (26 g) was refractionated using the vacuum column chromatography method with the eluent ethyl acetate: acetone (increasing polarity) and eight subfractions (M3A-M3H) were obtained. The M3G subfraction (1.6 g) was recrystallized using the solvent ratio *n*-hexane: acetone and a white solid (448.4 mg) was obtained as compound 1^18^. Structure elucidation of compound 1 was carried out using FTIR, ^1^H NMR, ^13^C NMR, DEPT 135 and 2D NMR (HSQC and HMBC) instruments.

### Statistical analyses

Extracts and the compound 1 that show the highest inhibitory activity of DPPH, ABTS, and α-glucosidase enzyme activity (%) will be evaluated to determine the IC_50_ value (μg/mL). This value was obtained by plotting the concentration of the test sample against antioxidant and antidiabetic activity in percentage (%) using linear regression. Experiment for antioxidant and antidiabetic activity was carried out in triplicates and the data were reported as the formula mean + standard deviation.

## Results

### Extraction

The crude *n*-hexane, ethyl acetate, dichloromethane and methanol extracts from *S. jamaicensis* leaves were monitored for spot profiles using thin-layer chromatography under UV light. During monitoring, the methanol extract of *S. jamaicensis* leaves showed dominant spots compared to other extracts, so the methanol extract was chosen for the fractionation of active compounds in *S. jamaicensis* leaves. Furthermore, the selection of extracts for the fractionation of active compounds in *S. jamaicensis* leaves was monitored from the bioactivity results of each extract.

### Antioxidant activity

**Figure 1:**
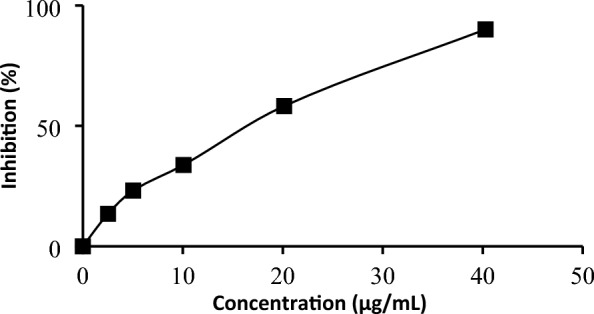
DPPH radical inhibition activity of methanol extract of *S. jamaicensis* leaves.

The DPPH radical scavenging activity of the four extracts, compounds and standard is presented in Table 1. The results showed that the methanol extract had good antioxidant activity against DPPH radical, as indicated by the high inhibitory percentage value of 90.34 ± 0.41% at 80.56 µg/mL with IC_50_ of 19.36 ± 0.34 µg/mL, as shown in Fig. 1. Furthermore, Trolox had an IC_50_ value of 17.10 ± 0.17 µg/mL. The ethyl acetate, dichloromethane and* n*-hexane extracts were observed to have less activity because they were not completely dissolved during the test.

**Table 1:** Antioxidant and α-glucosidase activities of extracts and isolated compounds from *S. jamaicensis.*

Samples	DPPH	ABTS	α-Glucosidase inhibition
	IC_50_ (μg/mL)	IC_50_ (μg/mL)	IC_50_ (μg/mL)
Extracts of *S. jamaicensis*			
*n*-hexane	> 550	202.25 ± 4.67	> 1250
Dichloromethane	> 550	85.68 ± 2.00	> 1250
Ethyl acetate	> 550	54.26 ± 0.29	> 550
Methanol	19.36 ± 0.34	27.37 ± 0.11	217.03 ± 16.00
The compound isolated from *S. jamaicensis*			
6β-hidroksipolamiide	> 550	393.25 ± 69.25	537.79 ± 45.04
Standard			
Trolox	17.10 ± 0.17	11.97 ± 0.25	–
Acarbose	–	–	2.94 ± 0.99

**Figure 2:**
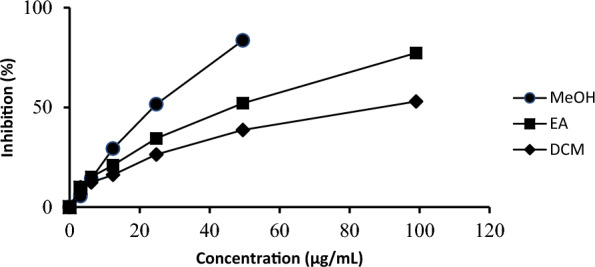
ABTS radical inhibition activities of various extracts: MeOH (methanol); EA (ethyl acetate), and DCM (dichloromethane) from *S. jamaicensis* leaves.

The methanol, ethyl acetate and dichloromethane extracts showed good inhibitory activity at a concentration of 99.00 µg/mL with IC_50_ values of 27.37 ± 0.11, 54.26 ± 0.29 and 85.68 ± 2.00 µg/mL, respectively, against ABTS cation radical (Fig. 2). Trolox as the positive control had an IC_50_ value of 11.97 ± 0.25 µg/mL in this study. Based on the preliminary results, the methanol extract can be used to recover compounds with antioxidant activity. Therefore, methanol was used as a solvent in the extraction process of *S. jamaicensis* leaves samples.

### Alpha-glucosidase inhibitory activity

**Figure 3:**
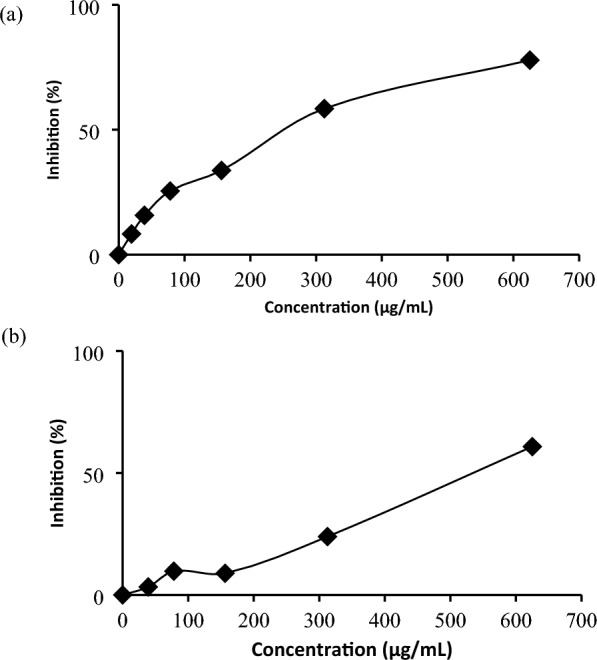
Inhibition of α-glucosidase of (**a**) the methanol extract, and (**b**) 6β-hydroxyipolamiide.

Evaluation of α-glucosidase activity showed that the methanol extract had the best inhibitory activities compared to ethyl acetate, dichloromethane and *n*-hexane extracts. The methanol extract had an inhibition percentage of 77.86 ± 3.01% with an IC_50_ value of 217.03 ± 16.00 µg/mL, while the 6β-hydroxyipolamiide showed 60.68 ± 1.46% at a concentration of 625 μg/mL (Fig. 3).

### Fractionation and elucidation

**Figure 4:**
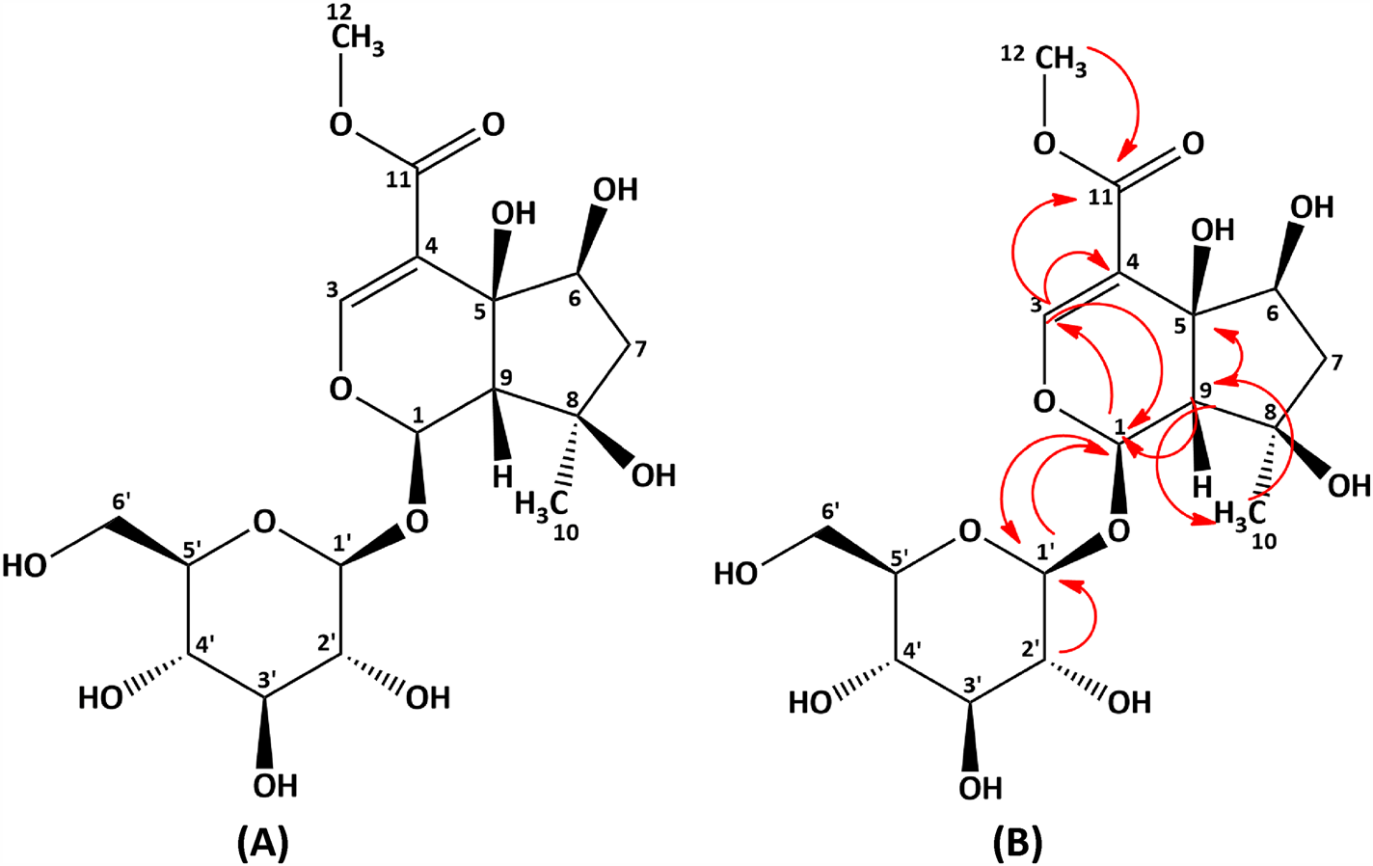
Structure (**A**) and HMBC (**B**) of 6β-hydroxyipolamiide.

The IR spectrum shows strong absorption bands at frequencies 3394 and 1697 cm^−1^ which indicate the presence of hydroxyl (–OH) and carbonyl (C=O) groups respectively. Apart from that, the presence of the CH sp^3^ group is also shown in the absorption band at 2904 cm^−1^. H-NMR and C-NMR data are shown in Table 2. ^1^H-NMR spectrum shows *δ*_H_ 2.02 (2H, m) and 0.96 (3H, s) ppm which indicates the presence of proton signals from methylene (CH_2_) and methyl groups (CH_3_). In addition, the typical proton signal of glycoside compounds also appears at shift 4.346 (1H, d), 3.65 (2H, m), 3.11 (1H, t), 3.04 (1H, t), 3.02 (1H, m) and 2.91 (1H, t) ppm. The typical proton signal of glycoside compounds also appears in the ^13^C-NMR spectrum at shift 98.4, 73.4, 76.5, 70.2, 77.3 and 61.5 ppm. There is a carbonyl group as evidenced by the signal at a shift of 166.3 ppm. Compound 1 has a melting point of around 130–131 °C. Based on the elucidation results, compound 1 was identified as 6β-hydroxyipolamiide which was compared with previous research^19^. The fractionation and elucidation of this compound have been previously reported by Yuliana et al.^18^. The structure and HMBC of the 6β-hydroxyipolamiide compound can be seen in Fig. 4.

**Table 2:** NMR spectroscopic data of 6β-hydroxyipolamiide^18^.

Position	*δ*_H_	*δ*c	HMBC
1	5.62 (1H, s)	93.2	3, 1’
3	7.31 (1H, s)	151.1	1, 4, 11
4	–	114.7	–
5	–	70.5	–
6	3.29 (1H, t)	73.4	–
7	2.02 (2H, m)	40.3	–
8	–	77.8	–
9	2.26 (1H, s)	61,2	1, 10, 5
10	0.96 (3H, s)	23.8	9
11	–	166.3	–
12	3.60 (3H, s)	51.3	11
1’	4.36 (1H, d)	98.4	1
2’	2.91 (1H, m)	73.4	1’
3’	3.11 (1H, m)	76.5	–
4’	3.04 (1H, m)	70.2	–
5’	3.02 (1H, m)	77.3	–
6’	3.65 (2H, m)	61.5	–

The compound 6β-hydroxyipolamiide had a relatively low antioxidant activity, as the highest concentration of each test method produced an inhibition percentage of < 50% compared to Trolox with IC_50_ of 11.97 ± 0.25 µg/mL. These findings showed that it had a low potential for usage as a source of natural antioxidants. 6β-hydroxyipolamiide had an IC_50_ of 537.79 ± 45.04 µg/mL (1.28 mM), while acarbose (positive control) showed 2.94 ± 0.99 µg/mL.

## Discussion

### Antioxidant activities of *S. jamaicensis*

*S. jamaicensis* is a plant from the *Verbenaceae* family, which was often found in tropical regions of Indonesia and had been used as a traditional diabetes medicine for decades. Several studies showed that it can cure allergies, respiratory conditions, coughs, colds, fever and digestive complications^11, 15^. In rural African, the leaves decoction was used for the treatment of dysentery or diarrhea^20^. Furthermore, it served as an anti-inflammatory, anti-malaria, anticholinesterase, anti-arthritic^17, 21^, anthelmintics^22^, antidiarrheal^23^ and immunomodulatory effect in the Caribbean and other tropical countries^24^. The secondary metabolites contained in this plant were reported to be responsible for these bioactivities. In this study, antioxidant activity of compounds isolated from *S. jamaicensis* was reported for the first time. Antioxidants are compounds with the ability to inhibit components produced from chemical reactions involving free radicals. Furthermore, free radicals can originate from sources outside the body (exogenous), such as air, water pollution, cigarette smoke, alcohol, heavy metals, medicines, solvents and radiation industries. They can also be produced inside the body due to inflammation, mental stress, excessive exercise, cancer and aging caused by auto-oxidation or molecular inactivation. The presence of excess free radicals caused oxidative stress, which can trigger various diseases, including diabetes, Alzheimer's, asthma, cataracts and cancer^3, 25^.

The antioxidant activities of the extracts and compounds were also measured using the DPPH and ABTS methods. DPPH was a stable and commercially available radical with a high sensitivity. It showed a strong maximum absorption at 517 nm with color changes from purple to yellow after the absorption of hydrogen from antioxidants. The results showed that the level of antioxidant effect was proportional to the loss of DPPH**·** absorption in the UV–vis spectrophotometer^26–30^. Furthermore, ABTS test involved the formation of ABTS + **·** cation radical due to the oxidation reaction with potassium persulfate. ABTS + cation radical absorbed a wavelength of 734 nm in the green or blue spectra, which were formed due to the loss of one electron on ABTS nitrogen atom. The presence of antioxidants led to the substitution of nitrogen atoms with hydrogen, leading to decolorization. The antioxidant effects were reported to be dependent on the activity and concentration levels of the test sample^31, 32^. Several studies showed that the methanol extract of *S. jamaicensis* had good antioxidant effects, but was lower compared to the standard. Similar results were obtained in this study, where the isolated compounds from *S. Jamaicensis*, namely 6β-hydroxyipolamiide, had antioxidant activity. However, the effects of these compounds were lower compared to the positive control of gallic acid and Trolox.

### α-Glucosidase inhibitory activity of *S. jamaicensis*

Some species of *Stachytarpheta* have been reported to have potential as antidiabetic agents. Diabetes is a complex disease caused by hyperglycemia due to impaired insulin secretion and/or damaged insulin action. Furthermore, type 2 diabetes was commonly found among adults aged > 45 years. It was often caused by increased demand for insulin by the body accompanied by insufficient production levels by the pancreas. This condition can also be triggered by several factors, including the environment, genetics and lifestyle. Type 2 diabetes was characterized by insensitivity to insulin and a decrease in production, which ultimately caused pancreatic beta cell failure. This can lead to a decrease in glucose transport to important organs in the body, such as the liver and muscles^33^.

The common treatment option for type 2 diabetes was the administration of an α-glucosidase inhibitor, an enzyme found on the border of the small intestine. These inhibitors have been reported to have the ability to delay the absorption of complex carbohydrates, thereby inhibiting glucose production and decreasing serum levels^34^. This chain of action can be triggered by antidiabetic plants^6, 28–30, 35^ containing secondary metabolites. The constituent metabolites can restore the function of pancreatic tissue by increasing insulin production and inhibiting intestinal absorption of glucose. Studies on the antidiabetic effect of the methanol extract from *S. jamaicensis* showed that 6β-hydroxyipolamiide had antidiabetic activity. However, their effects were lower compared to the positive control, namely acarbose, which was a commercial antidiabetic drug.

## Conclusion

*S. jamaicensis* of East Indonesian origin was often used for the treatment of various diseases, specifically diabetes. This is the first study to report the antioxidant and antidiabetic activity of chemical constituents isolated from the plant. The compound of 6β-hydroxyipolamiide was successfully isolated from the methanol extract of *S. jamaicensis* leaves. Besides that, the methanol extract of *S. jamaicensis* leaves can inhibit DPPH, ABTS and α-glucosidase enzyme activity compared to other solvent extracts and the compound of 6β-hydroxyipolamiide. However, the compound of 6β-hydroxyipolamiide inhibited the α-glucosidase enzyme activity compared extract of *S. jamaicensis* leaves. Therefore, further research regarding the identification of other active compounds in *S. jamaicensis* leaves is required to support the bioactivity of this plant.

### Supplementary Information

Supplementary Information.

## Abbreviations

DPPH: 2,2-Diphenyl-1-picrylhydrazyl
ABTS: 2,2’-azinobis- (3-ethylbenzothiazoline-6-sulfonic acid)
IC_50_: Half inhibitory concentration
FTIR: Fourier transform infrared
NMR: Nuclear magnetic resonance
DEPT: Distortionless enhancement by polarization transfer
HSQC: Heteronuclear single quantum coherence
HMBC: Heteronuclear multiple bond correlation
DNA: Deoxyribonucleic acid
DMSO: Dimethylsulfoxide
K_2_S_2_O_8_: Potassium peroxydisulphate
RPM: Revolutions per minute
UV: Ultraviolet
MeOH: Methanol
DCM: Dichloromethane
EA: Ethyl acetate
DPPH•: Radical 2,2-Diphenyl-1-picrylhydrazyl
ABTS+: Kation 2,2’-azinobis- (3-ethylbenzothiazoline-6-sulfonic acid)
