# Supplementary material for: Antioxidant and α-glucosidase inhibitory activities of compound isolated from Stachytarpheta jamaicensis (L) Vahl. leaves
Source: Sci Rep. 2023 Oct 30;13:18597. doi: 10.1038/s41598-023-45357-z (PMC10616201; doi:10.1038/s41598-023-45357-z)
Supplement: Supplementary file 1 — Supplementary Information. [file 41598_2023_45357_MOESM1_ESM.docx]

**SUPPLEMENTARY DATA : Spectroscopic Data of 6β-hydroxyipolamiide**

1. **FTIR Spectrum**

**
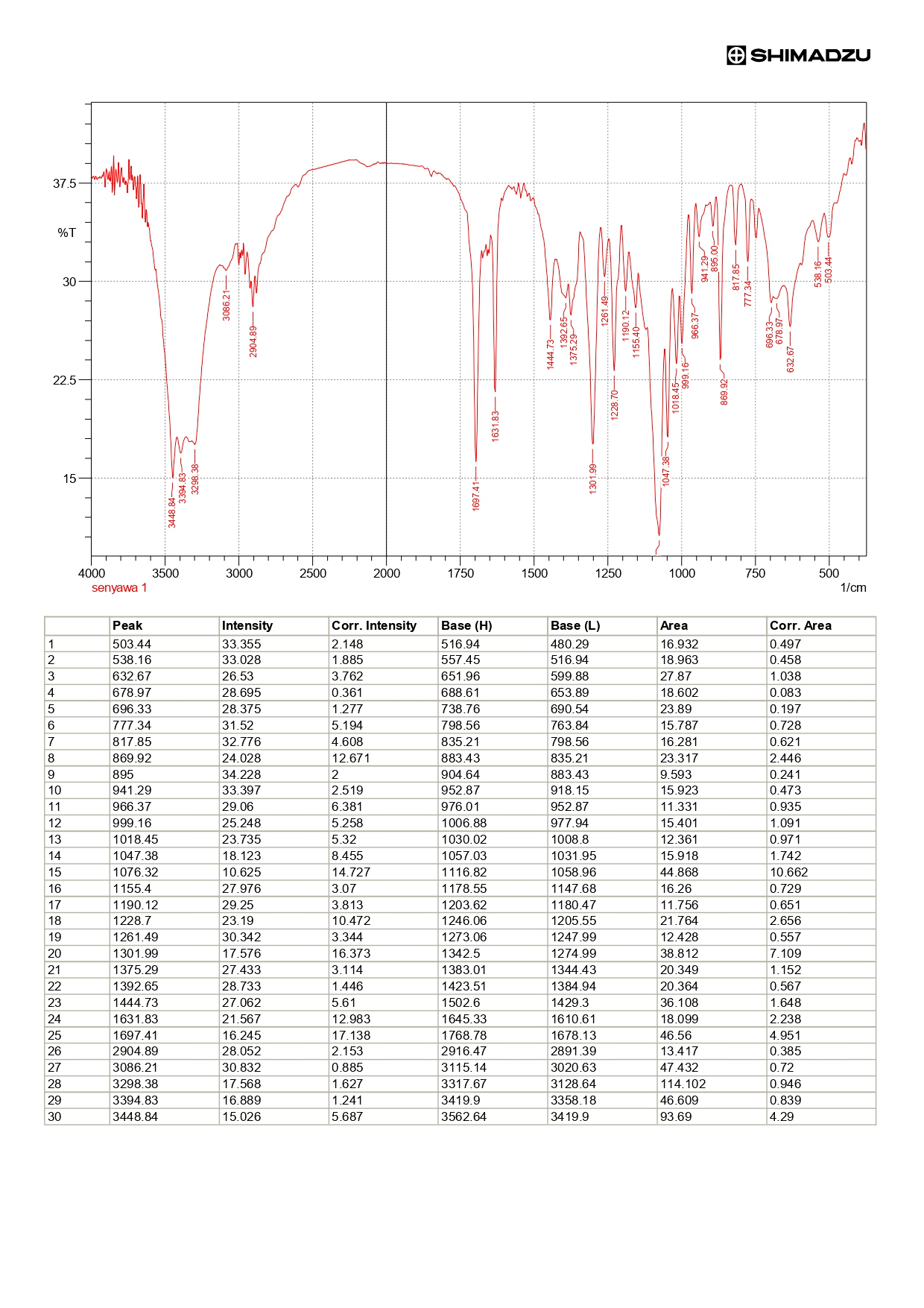
**

**
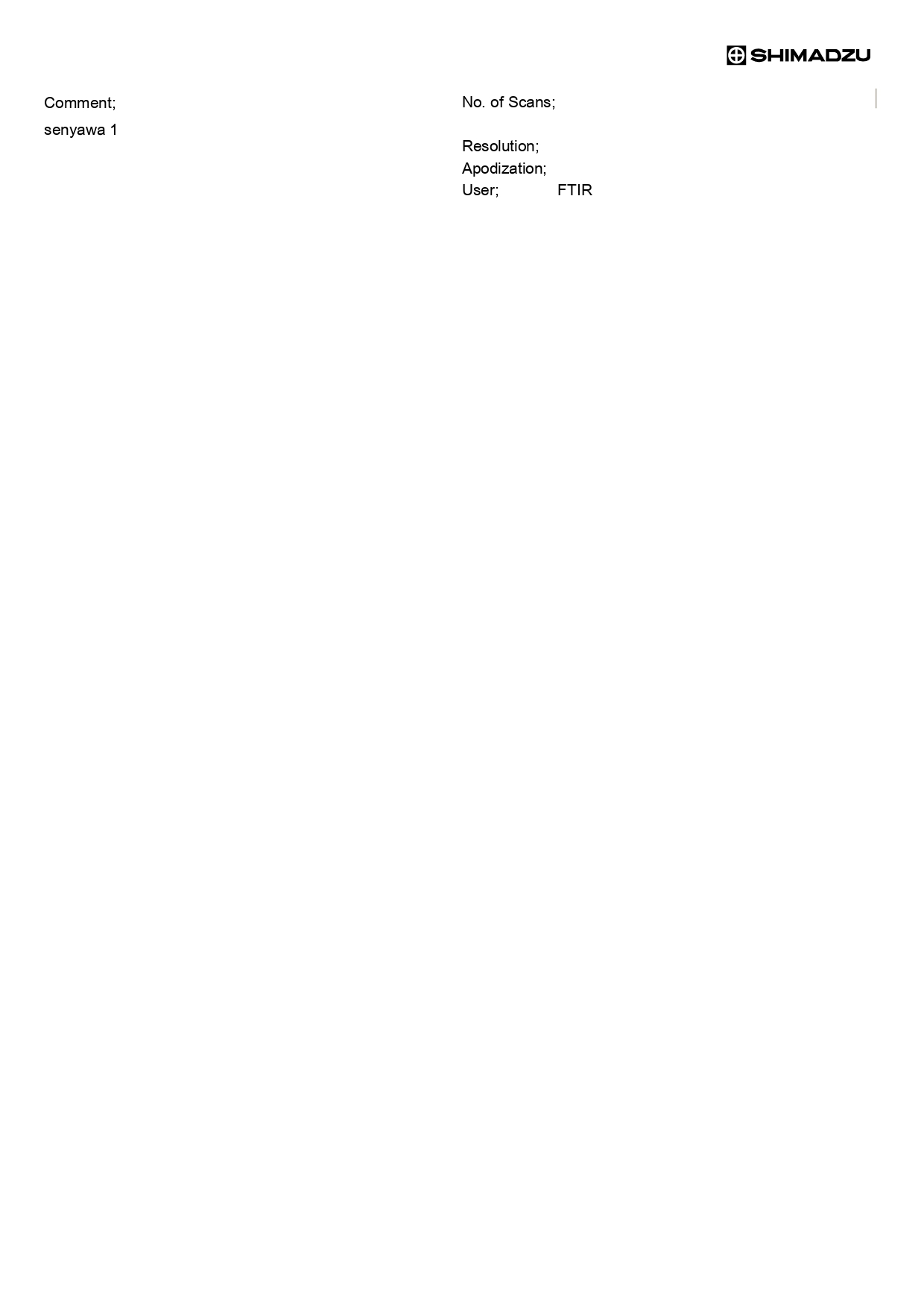
**

1. **2. ^1^H NMR Spectrum**

**
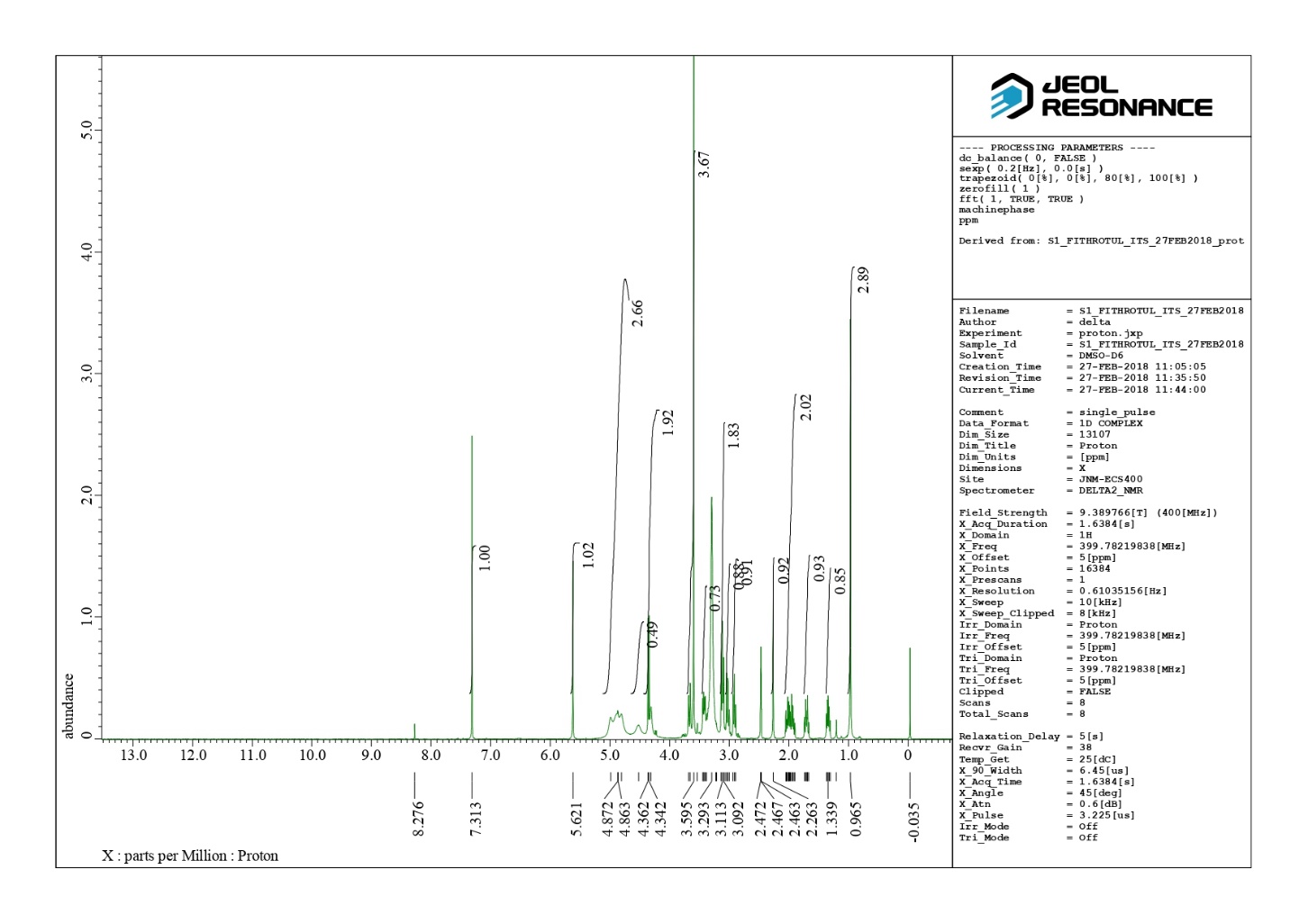
**

1. **^13^C NMR Spectrum**

**
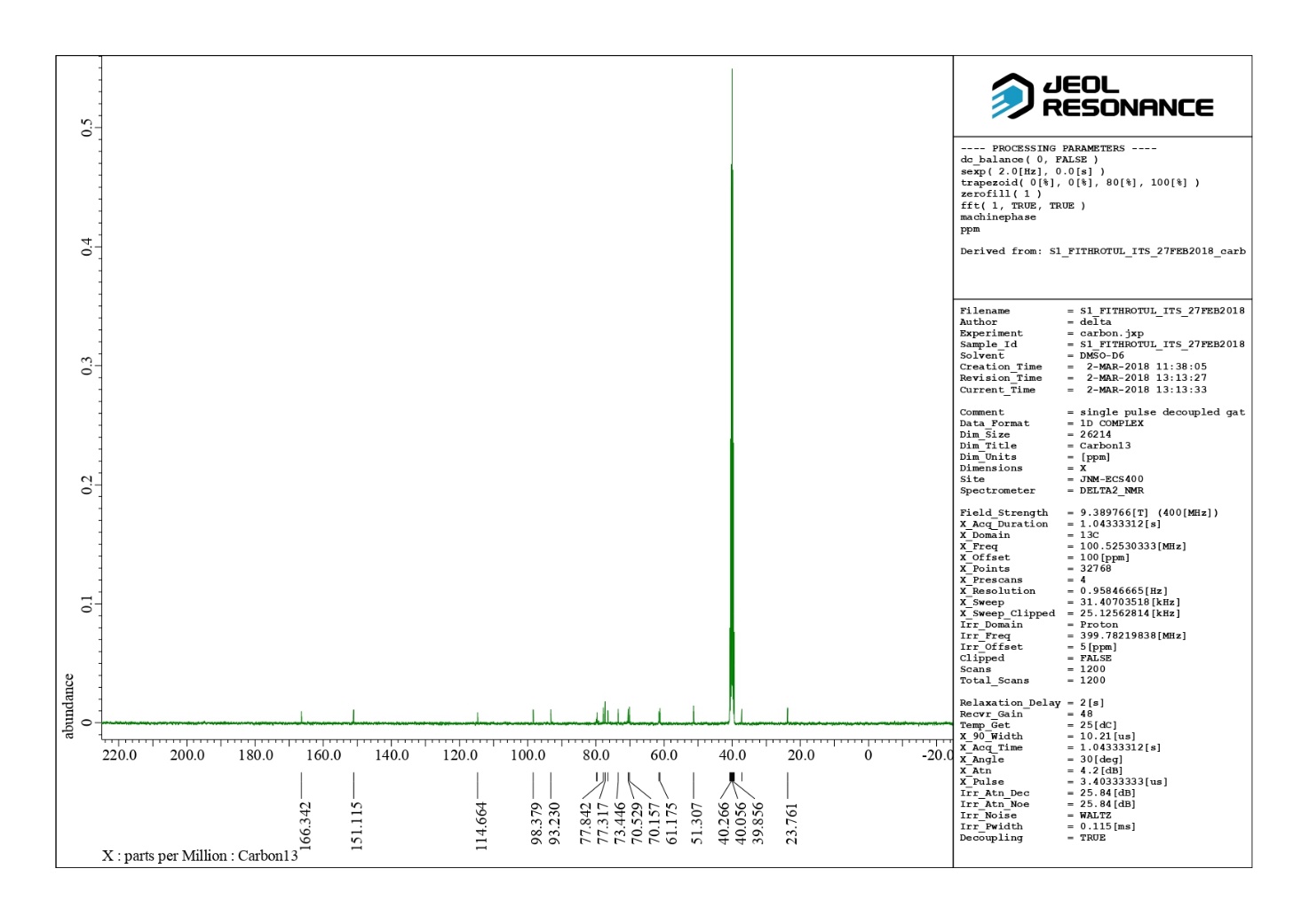
**

1. **DEPT 135 Spectrum**

**
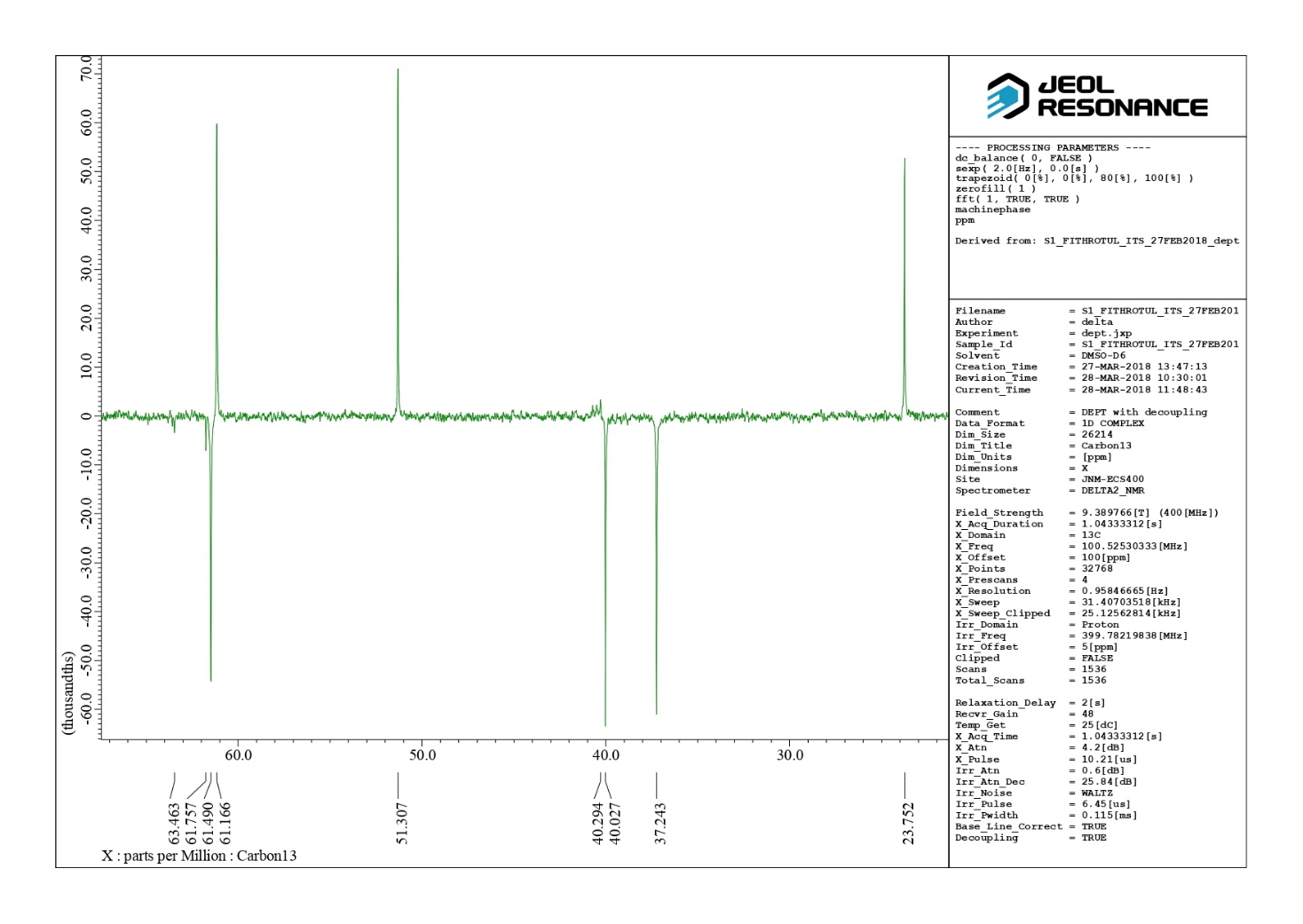
**

**
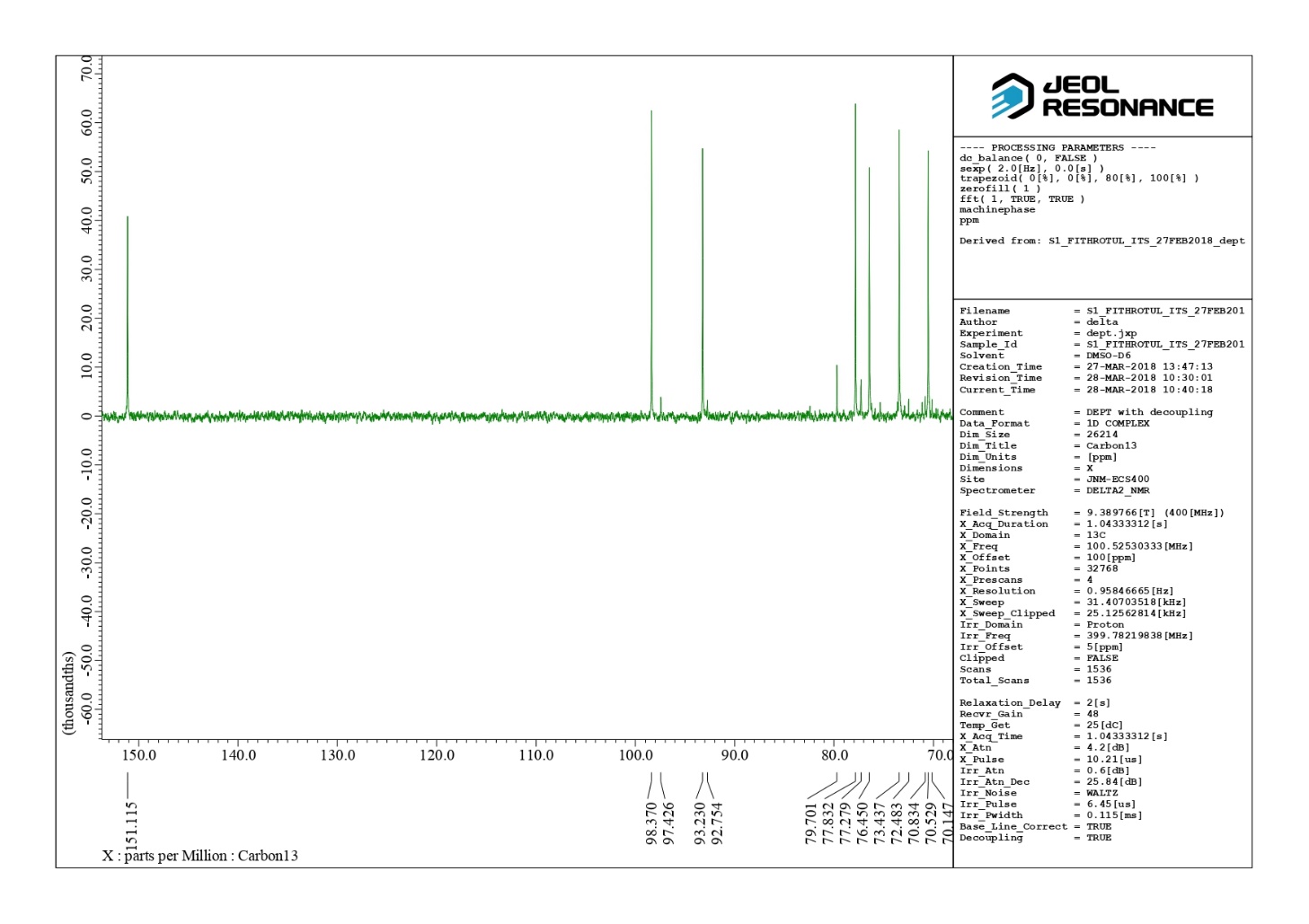
**

1. **HSQC Spectrum**

**
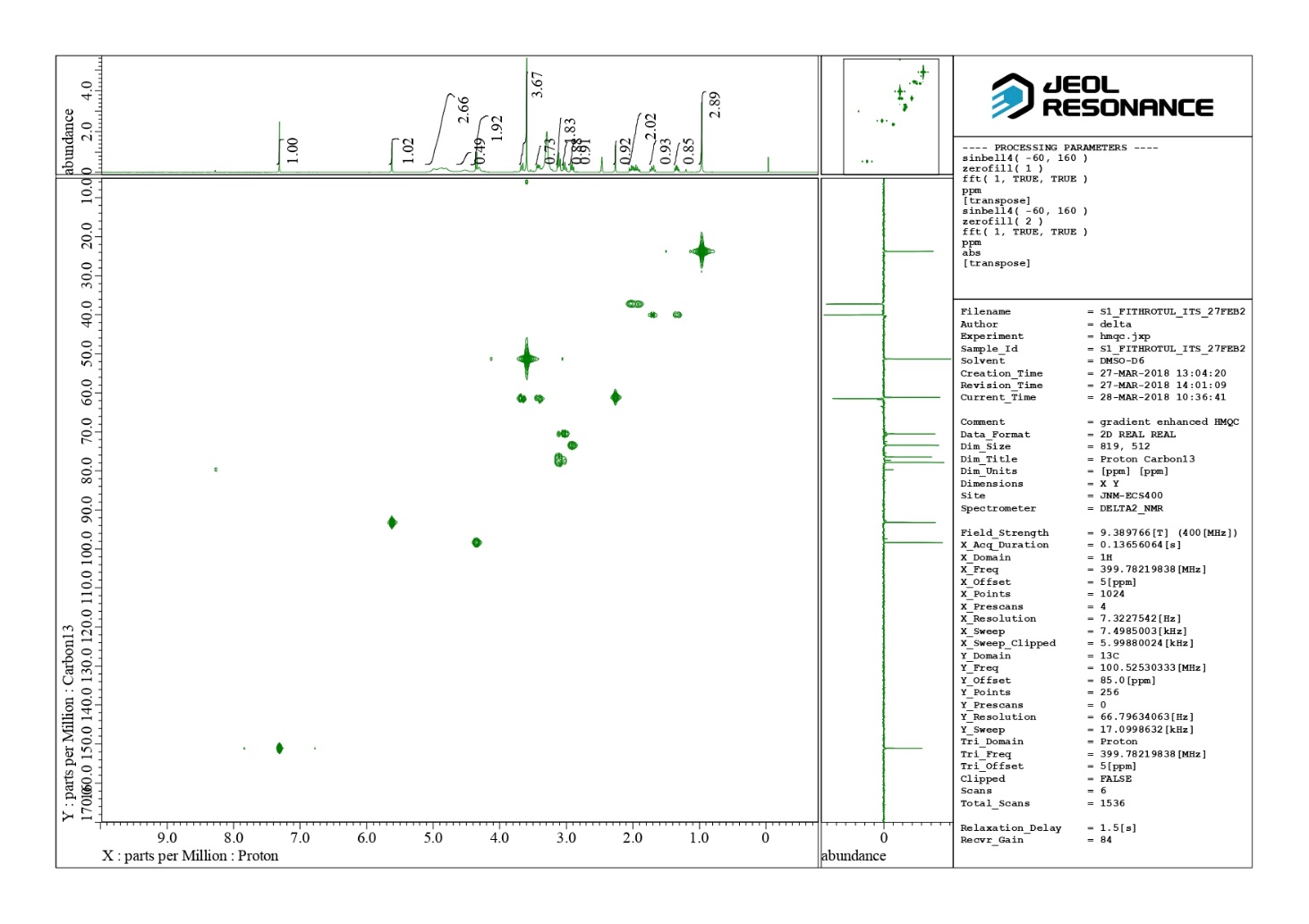
**

1. **HMBC Spectrum**

**
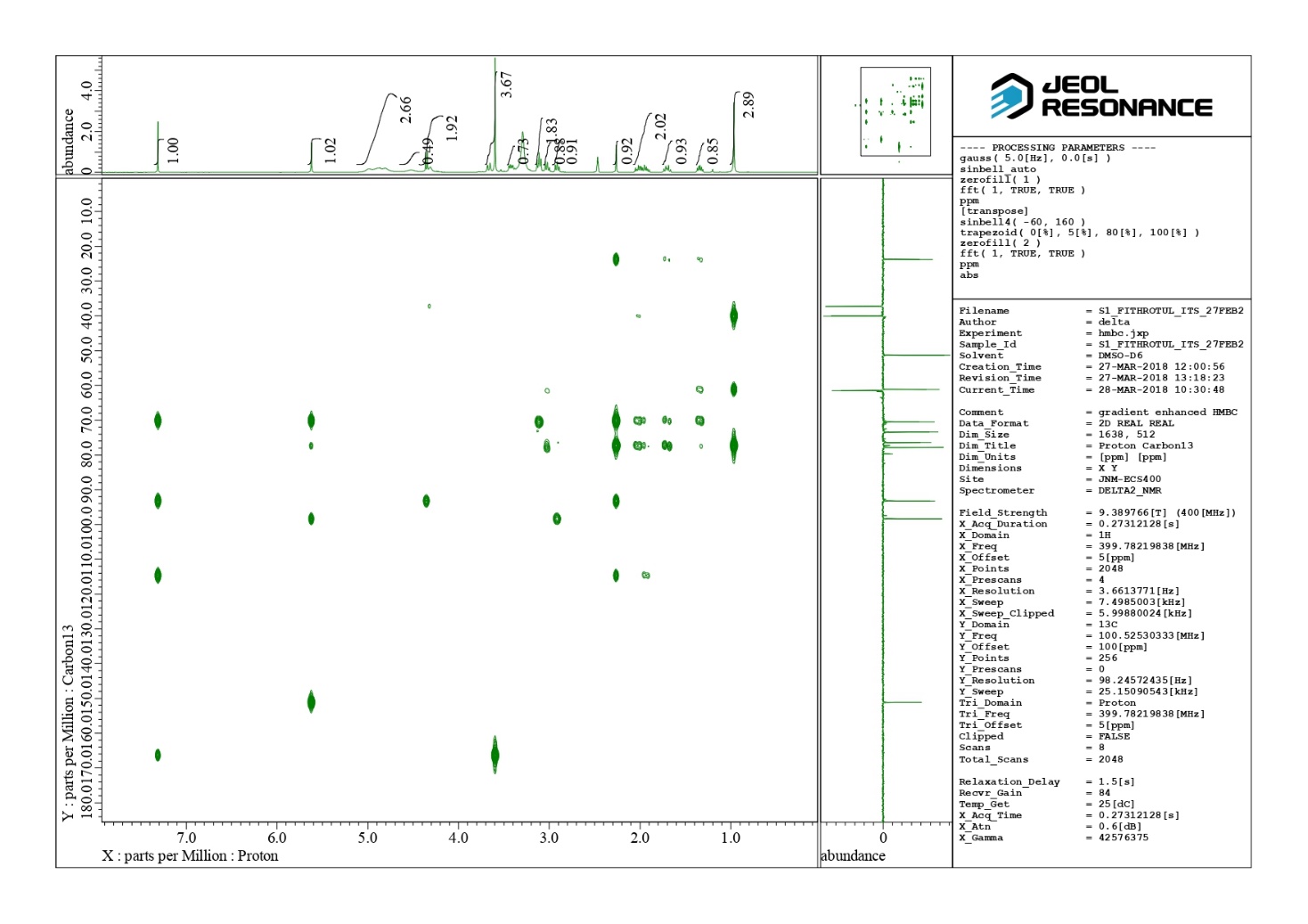
**
